# Hepatic encephalopathy treatment after transjugular intrahepatic portosystemic shunt: a new perspective on the gut microbiota

**DOI:** 10.3389/fmed.2025.1423780

**Published:** 2025-03-07

**Authors:** Xiaotong Xu, Tong Zhu, Changyou Jing, Minjie Jiang, Yunlai Fu, Fang Xie, Qinghua Meng, Jianjun Li

**Affiliations:** ^1^Department of Oncology, Beijing Youan Hospital, Capital Medical University, Beijing, China; ^2^Beijing Institute of Hepatology, Beijing Youan Hospital, Capital Medical University, Beijing, China; ^3^Interventional Therapy Center for Oncology, Beijing Youan Hospital, Capital Medical University, Beijing, China

**Keywords:** transjugular intrahepatic portosystemic shunt, hepatic encephalopathy, gut microbiota, probiotics, treatment, prevention

## Abstract

Transjugular intrahepatic portosystemic shunt (TIPS) placement alleviates portal hypertension symptoms. Hepatic encephalopathy (HE) is a common complication of TIPS, impacting patient quality of life and the healthcare burden. Post-TIPS HE is associated with portosystemic shunting, elevated blood ammonia levels, and inflammation. Increasing attention has been given to the liver and intestinal circulation in recent years. An imbalance in intestinal microecology plays a role in the occurrence of HE and may be a new target for treatment. This review discusses the causes, diagnosis, and treatment strategies for post-TIPS HE and focuses on exploring treatment strategies and their relationships with the gut microbiota, suggesting an innovative approach to address this complication.

## Introduction

Transjugular intrahepatic portal system shunt (TIPS) is one of the main methods used to reduce portal vein pressure and works by establishing a new channel between the portal vein and hepatic vein, which can quickly reduce portal vein pressure, achieve hemostasis, and relieve ascites (1–4). Compared with abdominal paracentesis, TIPS significantly enhances transplant-free survival in cirrhosis patients suffering from refractory ascites. It also diminishes the likelihood of recurrent ascites and hepatorenal syndrome. However, TIPS is associated with an increased risk of developing hepatic encephalopathy (HE) (5). The gut–liver axis has emerged as a focal point in chronic liver disorders, prompting more research into the role of the gut microbiota in liver cirrhosis (6). In individuals with liver cirrhosis, changes in the structure and function of the gut microbiota are closely tied to clinical prognosis (7–9). The gut microbiota is closely related to the occurrence of HE (10–13). At the same time, changes in portal pressure can also affect the gut microbiota (14–17). Intestinal congestion in patients with portal hypertension not only affects the absorption of nutrients but also leads to local inflammatory reactions, damages the intestinal barrier function, affects the intestinal microenvironment, and thus affects the composition of the intestinal microbiota. TIPS can improve the intestinal microenvironment to a certain extent by reducing portal pressure, reducing intestinal congestion, alleviating local inflammation, and repairing the intestinal barrier. Studies have shown that the better the intestinal microbiota recovers after TIPS, the lower the incidence of HE (18).

HE is a common complication after TIPS that affects the quality of life of patients and their families. Therefore, this review aims to summarize the relevant risk factors for HE occurrence after TIPS, the role of the gut microbiota in diagnosing HE and predicting patient prognosis, and prevention and treatment strategies.

## Definition, classification, and adverse effects of HE

HE is a complex neuropsychiatric syndrome caused by acute and chronic liver dysfunction or portal-systemic shunt abnormalities characterized by metabolic disorders with varying degrees of severity (19). In general, HE can be divided into three types depending on the type of liver disease: type A (caused by acute liver failure), type B (associated with portocaval shunt), and type C (associated with chronic liver injury such as cirrhosis with the presence of portosystemic shunts) (19). The West Haven HE classification standard (0–4 levels) (20) has been widely utilized. This classification system includes non-HE, minimal hepatic encephalopathy (MHE), HE Level 1, and HE Levels 2–4. The first three grades are collectively referred to as covert hepatic encephalopathy (CHE). Patients with CHE have only mild cognitive difficulties such as decreased attention, memory, and delayed responses. HE Levels 2–4 are collectively referred to as overt hepatic encephalopathy (OHE). Patients with OHE may experience personality changes, comas, or other neurological abnormalities. The diagnosis of HE relies mainly on symptoms and neuropsychological tests, while excluding altered mental status caused by other reasons. MHE can affect the clinical prognosis and may progress to OHE (21). MHE may persist even after recovery (22). In addition to increasing falls, fractures, and traffic accidents, HE can also increase the length of hospital stays, rate of rehospitalization (21), and mortality (23, 24), affecting patient prognosis (25) and the quality of life of patients’ families (26) and leading to an increasing healthcare burden (27).

## Incidence rate and related factors of post-TIPS HE

**Figure 1:**
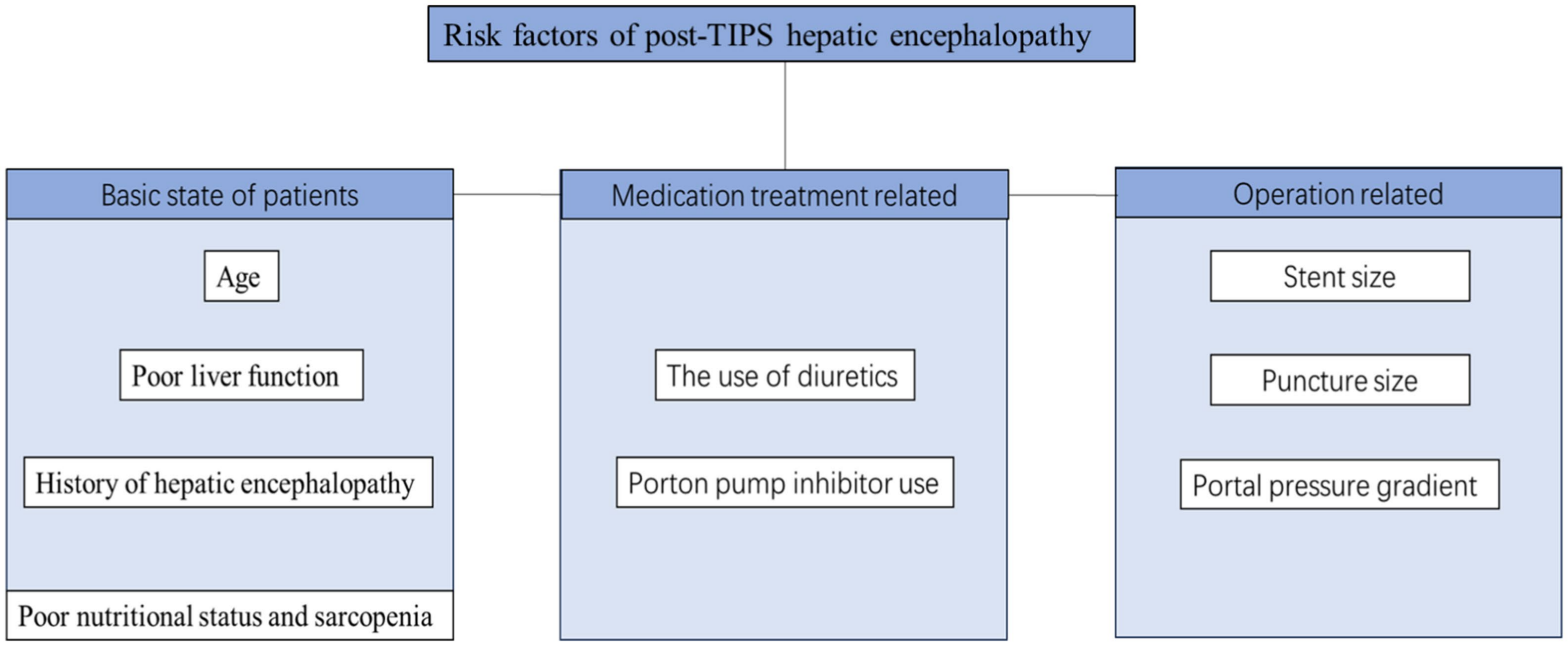
Common risk factors for post-TIPS HE.

Transjugular intrahepatic portosystemic shunt (TIPS) placement is one of the main methods used to treat portal hypertension. However, HE is one of the most frequent postoperative complications after TIPS (28). The incidence of HE after TIPS varies due to factors such as previous history of HE, puncture site, and follow-up time with an average incidence of 20–50% (29–31). In a retrospective study of 75 patients with no previous episodes of HE, the incidence of HE at 6 months after TIPS was 36% (30), whereas it was 27% at 12 months. A study involving 82 patients with liver cirrhosis with previous HE reported that the incidence of OHE after TIPS at 6 months was 43% (29). Among patients who underwent right portal TIPS within 1 year, 46.7% had HE, whereas 26.6% of patients who underwent left portal TIPS had HE (32). HE after TIPS is related to the establishment of a shunt channel between the portal and central veins during surgery, which limits the ability of the liver to detoxify intestinal toxins. When the toxin crosses the blood–brain barrier, they lead to impaired brain function, resulting in new or exacerbations of existing HE. Many related factors may affect the occurrence of post-TIPS HE (Figure 1).

### The basic state of patients: age, history of HE, liver status, and nutritional status are associated with post-TIPS HE

Age (sHR 1.05, CI 1.02–1.08, *p* = 0.002), Child-Pugh score (sHR 1.29, CI 1.06–1.56, *p* = 0.01), and CHE (sHR 3.16, CI 1.43–6.99, *p* = 0.004) have been reported to be associated with post-TIPS HE (29). OHE after TIPS developed significantly more frequently in patients with a history of OHE or MHE (33). A meta-analysis (34) also revealed that patients with HE before TIPS or a higher Child-Pugh class or score had an increased risk of post-TIPS HE. A recent study revealed (35) that liver function deteriorated initially after TIPS, followed by recovery. Recovery of liver function at 3 months was associated with reduced OHE. Nutritional status is related to liver function, and sarcopenia can directly reflect nutritional status, while muscles participate in ammonia detoxification. Sarcopenia is a common complication of liver cirrhosis, is related to the severity of liver disease, and increases the incidence of other liver disease complications, including HE (36). In a prospective study (37) of 46 patients with cirrhosis, sarcopenia (sHR 31.3, CI 4.5–218.07, *p* < 0.001) was independently associated with the development of HE after TIPS. The mechanism underlying the relationship between sarcopenia and HE may be related to decreased ammonia detoxification secondary to sarcopenia. Because muscles are another important site for ammonia metabolism, creating glutamine through glutamine synthase, muscle loss may lead to a decrease in the ability to clear ammonia in the body, thereby increasing the risk of HE (38). There is a complex interaction between sarcopenia and HE (39), so it is not clear which is the cause and which is the effect of sarcopenia and HE. Previous studies have suggested that sustained high blood ammonia levels may increase the levels of muscle growth inhibitors while stimulating autophagy, leading to muscle protein synthesis disruption and muscle atrophy (40, 41). However, a recent meta-analysis (36) revealed via multivariate analysis that HE did not increase the risk of sarcopenia (2.14, 95% CI 0.56–8.16) (*I*^2^ = 62%, *p* = 0.07).

### Medication treatment: diuretic and proton pump inhibitors may increase the occurrence of HE after TIPS

Dilutional hyponatremia commonly develops in patients with advanced cirrhosis and portal hypertension when patients have insufficient oral sodium intake and excessive use of diuretics, causing brain edema and a series of neurological manifestations. One study aimed to assess the effect of hyponatremia on the development of OHE within 1 week of TIPS and reported that the odds ratio for developing HE with hyponatremia (pre-TIPS Na <135 mEq/L) was 8.6 (42). Proton pump inhibitors (PPIs) are commonly used to alleviate gastric acid discomfort in patients and reduce the risk of bleeding. However, PPI lowers the pH of the gastrointestinal tract by inhibiting gastric acid secretion. While this may lead to more ammonia being absorbed into the bloodstream through the intestine, it weakens the gastric acid barrier, causing bacterial translocation and affecting the composition of the gut microbiota. This is beneficial for the growth of urease-producing bacteria, thus increasing the risk of developing HE (43, 44). Research has shown that patients who receive PPI treatment during TIPS exhibit a significantly greater incidence of post-TIPS HE than patients without PPI treatment do (30.4% vs. 11.7%, *p* < 0.001). The incidence of HE after TIPS increased in a dose-dependently manner with the use of PPI. Patients administered 40 mg PPIS daily presented a notably greater incidence of post-TIPS HE than those on a 20 mg PPI daily dose (30.9% vs. 10.0%, *p* = 0.028) (45).

### Operation: stent diameter, puncture site, and changes in the portal vein pressure gradient are related to post-TIPS HE

With respect to stent diameter, a randomized controlled trial (46) revealed that the incidence of HE was similar in groups with an 8 mm and 10 mm stent diameter. However, a meta-analysis (47) aimed at exploring the optimal diameter of TIPS suggested that for Asians, the use of a stent with a diameter of 8 mm was beneficial for reducing the incidence of postoperative HE (OR = 0.49, CI 0.27–0.87, *p* = 0.02). Another meta-analysis revealed that (48) the rate of postoperative HE was significantly lower in the group with the puncture site in the left portal vein group than in the group with the site in the right portal vein (5.7% vs. 18.1%, OR 0.19, *p* < 0.00001). The blood flow inside the stent corresponds to the degree of decrease in the portosystemic gradient (PSG), and the greater the decrease in the PSG before and after TIPS, the greater the degree of diversion into the body is. The greater the venous blood flow is, the greater the likelihood of developing HE (49). According to reports, TIPS reducing the hepatic venous pressure gradient by >9–10 mmHg or > 60% increases the risk of HE after TIPS. A study conducted in 1280 patients who underwent TIPS due to refractory ascites or variceal bleeding revealed that a one-third reduction in PSG can reduce the risk of HE and liver function damage and achieve good clinical results (50).

## Intestinal microbiota may be a potential mechanism for post-TIPS HE

Most patients undergoing TIPS treatment have a foundation for liver disease, such as cirrhosis. On the one hand, the efficiency of ammonia metabolism by damaged liver cells is reduced; on the other hand, due to the placement of the stent, the amount of ammonia entering the brain tissue further increases, saturating the glutamine metabolism pathway (51–53). Excessive glutamine can damage the morphology and function of astrocytes, thereby affecting the integrity of the blood–brain barrier. Modified astrocytic function ultimately results in disrupted neuroglial interactions and an imbalance within the neurotransmitter system. This disruption affects synaptic plasticity and the functioning of cerebral oscillatory networks, creating a pathological setting that is indicative of HE (54, 55). The possible mechanism is that excessive glutamine is transported to the mitochondria and metabolized by glutaminase into glutamate and ammonia. Excess ammonia interferes with normal mitochondrial function, producing excessive reactive oxygen species and reactive nitrogen and inducing mitochondrial permeability transition (56, 57).

The ammonia toxicity theory is one of the main mechanisms of HE, and the ammonia produced by the intestinal flora is a key driving factor for HE (58). With the development of technologies such as metagenomics and a deeper understanding of the intestinal microbiota, increasing attention has been given to the role of the microbiota, inflammation, and metabolic pathways in the pathogenesis of HE, providing a new perspective for treatment (11, 59).

### Intestinal liver axis and liver diseases

The intestinal microecology is an ecosystem formed by the interaction between intestinal microorganisms and the human body. The balance of the intestinal microecology depends on the stable intestinal flora, normal intestinal mucosal barrier, and normal operation of related lymphatic tissues. The intestinal microbiota comprises bacteria, fungi, viruses, and protozoa. The intestine and liver are interconnected and interact through the biliary tract, portal vein, and systemic circulation, which is known as the intestine–liver axis (60). On the one hand, when liver function is impaired, bile acid synthesis is reduced, and the intestinal tract is more susceptible to competitive colonization by bacteria. Reduced albumin synthesis and portal hypertension lead to intestinal edema, resulting in excessive bacterial growth and damage to the intestinal mucosal barrier. On the other hand, when the intestinal microbiota is dysfunctional, the immune system can affect the degree of liver steatosis, inflammation, and fibrosis. As observed in non-alcoholic fatty liver disease, the abundance of bacteria in feces is independently associated with non-alcoholic steatohepatitis, and the abundance of *Ruminococcus* is independently associated with significant liver fibrosis (*F* ≥ 2) (61). The intestinal microbiota is closely linked to liver diseases (62). An increasing number of studies have reported different microbiota phenotypes in various liver diseases (60, 63, 64). Understanding the intestinal liver axis has led to the development of new diagnostic, prognostic, and therapeutic methods for liver diseases.

### The intestinal microbiota is related to post-TIPS HE

The occurrence of HE is closely related to disturbance of intestinal microbiota (10). Bajaj et al. (65) found that patients with HE had lower *Roseburia* and higher *Enterococcus*, *Veillonella*, *Megasphaera*, and *Burkholderia* abundances in the mucosal microbiome than non-HE patients did. The abundance of butyrate-producing bacteria increased in the intestinal mucosa of patients with cirrhosis without HE. Moreover, autochthonous genera (*Blautia*, *Faecalibacterium*, *Roseburia*, and *Dorea*) were associated with good cognition and decreased inflammation in patients both with and without HE, whereas *Enterococcus*, *Megasphaera*, and *Burkholderia* were associated with poor cognition and inflammation (65). However, there was no significant difference in the fecal microbiota between patients with cirrhosis with and without HE, suggesting that the fecal microbiota may have a smaller impact on immunity and overall health than the intestinal mucosal microbiota does. Another study (66) comparing the biological differences in stool microflora between patients with cirrhosis with and without MHE reported that the abundance of *salivary streptococci* in patients with MHE was significantly greater than that in patients without MHE (*p* = 0.030), and the change in the number of these bacteria was positively correlated with ammonia accumulation (*R* = 0.58, *p* = 0.003). Some studies found that Saboo et al. (67) have shown that the function and composition of the intestinal microbiota in cirrhosis patients with HE are related to sex. As the disease progresses in male patients with liver cirrhosis, the flora related to hormone metabolism changes, and the microbial composition is similar to that in female patients. In general (68), patients with liver cirrhosis have an imbalance in the intestinal flora, which is manifested mainly by an increase in bacteria that promote inflammation and ammonia production. As our understanding of the liver gut–brain axis increased, an increasing number of microbiota have been identified as contributing to HE.

The establishment of portal-systemic shunt channels reduces the detoxification effect of the liver against intestinal endotoxins, which may increase the incidence of HE after TIPS. It also improves portal hypertension, which can affect the composition of the intestinal microbiota to a certain extent and improve the intestinal microecology (14). The specific mechanism of post-TIPS HE, as well as whether the change in the intestinal flora is a concomitant phenomenon of HE or is involved in the occurrence of HE, remains to be further explored. There is an interaction between portal hypertension and the intestinal flora. On the one hand, portal hypertension can cause intestinal congestion, damage the intestinal barrier, and lead to intestinal flora translocation. On the other hand, flora translocation can also increase liver inflammation, damage liver function, and further increase portal vein pressure (14, 16), which can increase the occurrence of adverse events such as HE.

One study (69) divided patients into three groups (non-HE, MHE, and OHE) according to prognosis after TIPS to investigate the changes in the gut microbiota after TIPS in patients with MHE. After TIPS, the non-HE group presented significant increases in the native flora *Dialister*, *Coprococcus, Ruminococcaceae_uncultured*, *Flavonifractor*, and *Clostridium_sensu_stricto_1*, whereas the MHE group presented significant reductions in the abundance of the harmful flora *Granulicatella*, *Enterococcus*, *Streptococcus*, and *Rothia* and significant increases in the abundance of *Veillonella* and *Megasphaera* after TIPS, whereas the OHE group presented a significant increase in the abundance of *Veillonella* only after surgery. There was a significant difference in the changes in the gut microbiota after TIPS between patients with different prognoses. The increase in the abundance of native flora may influence the remission of MHE. Another study (18) aimed to evaluate alterations in the microbiota after TIPS and the relationship between these changes and HE. After TIPS, the autochthonous taxa increased, whereas the potential pathogenic taxa decreased in the non-HE group, and the autochthonous taxon *Lachnospiraceae* decreased in the HE group. The variations in five autochthonous taxa, namely, *Coprococcus*, *Ruminococcus*, *Blautia*, *Ruminococcaceae_uncultured*, and *Roseburia*, were negatively correlated with the severity of HE. The gut microbiota could be a promising potential biological target for screening suitable patients receiving TIPS and prevention and for the treatment of post-TIPS HE. Hong-Wei Zhao (70) reported that the abundance of gut microbiota at the phylum level did not differ between the HE group and the non-HE group after TIPS. However, the abundances of *Haemophilus* and *Eggerthella* increased, whereas those of *Anaerostipes*, Dialister, *Butyricicoccus*, and *Oscillospira* decreased in the HE group. The abundances of *Eggerthella*, *Streptococcus*, and *Bilophila* increased, whereas those of *Roseburia* and *Ruminococcus* decreased in the non-HE group, and the pathogenic genus *Morganella* appeared in the HE group but not in the non-HE group.

To date, few studies have focused on metabolic changes after TIPS. A recent Italian study (71) evaluated whether TIPS placement modified the gut microbiota composition and metabolic function. The results revealed that (71) abundance of *Flavonifractor* spp. increased (*p* = 0.049) after TIPS and the abundance of *Clostridiaceae* decreased (*p* = 0.024). No differences were detected in the short-chain fatty acid signature, whereas analysis of medium-chain fatty acid (MCFA) profiles revealed a decreased abundance of proinflammatory isohexanoic (*p* < 0.01), 2-ethylhexanoic (*p* < 0.01), and octanoic (*p* < 0.01) acids after TIPS. Correction of portal hypertension following TIPS resulted in modifications of the gut microbiota composition, which could be beneficial and reduce the levels of fecal proinflammatory MCFA. A non-targeted metabolomics study conducted in 22 patients with cirrhosis who underwent TIPS revealed that the placement of TIPS stents affects metabolomic changes, indicating that low levels of bile acids in peripheral blood after TIPS are associated with an increase in the severity of HE (72). Another study revealed that peptides, amino acids, and lipid metabolites significantly increased among the early postoperative metabolites of TIPS, mainly enriched in the pathway of amino acid metabolism. The most significant metabolite consumed was the lipid metabolite. Moreover, it was found that 9 portal vein metabolites had good predictive value in predicting liver function decline after TIPS, and 12 portal vein metabolites had moderate classification performance in predicting HE grade (73).

Changes in the intestinal microecology after TIPS may be related to decreased portal vein pressure and reduced intestinal congestion. Therefore, the intestinal flora before TIPS may be related to patient survival, and the degree of recovery of the intestinal flora after TIPS may be related to the incidence and severity of HE. Previous studies have shown that (74) the fecal flora is associated with the onset of HE in patients with liver cirrhosis and that the relative abundance of individual flora is associated with HE recurrence and overall survival during follow-up. Therefore, the intestinal microbiota may become a new marker for predicting HE after TIPS, but there is still little research on this topic. The role of the gut microbiota in the diagnosis and prediction of HE is shown in Table 1.

**Table 1:** Role of gut microbiota in diagnosing and predicting HE.

References	Subject	Sample	Method	Result	Clinical significance
HE and gut microbiota					
Bajaj et al. (65)	*N* = 60 patients with cirrhotic (*N* = 36HE vs. *N* = 24 non-HE) and controls (*N* = 17)	Sigmoid biopsies and Fecal samples	Multitag pyrosequencing	Between patients with/without HE patients, there was no difference in stool microbiota, but the mucosal microbiome was different with lower *Roseburia* and higher *Enterococcus, Veillonella, Megasphaera,* and *Burkholderia* abundance in HE.	The mucosal microbiota of patients with cirrhosis, especially those with HE, lack potentially beneficial autochthonous genera and have overgrowth of potentially pathogenic genera, which are associated with poor cognition and inflammation.
Bajaj et al. (68)	*N* = 24 patients with cirrhosis with HE *N* = 8 patients with cirrhosis without HE *N* = 10 control	Fecal samples	Multitag pyrosequencing	In the cirrhosis group, *Alcaligenaceae* and *Porphyromonadaceae* were positively correlated with cognitive impairment. *Fusobacteriaceae*, *Veillonellaceae*, and *Enterobacteriaceae* were positively and *Ruminococcaceae* was negatively related to inflammation.	Specific bacterial families (*Alcaligenaceae*, *Porphyromonadaceae*, and *Enterobacteriaceae*) are strongly associated with cognition and inflammation in HE.
Zhang et al. (66)	*N* = 26 patients with cirrhosis with MHE *N* = 26 MHE-matched normal relatives *N* = 25 patients with cirrhosis without MHE	Fecal samples	16S rDNA gene sequences	*Streptococcacea*e and *Veillonellaceae* increased in patients with cirrhosis with and without MHE, compared with normal individuals. *S. salivarius* was significantly higher in patients with cirrhosis with MHE and was positively correlated with ammonia accumulation.	Gut ammonia-increasing bacteria *S. salivarius* might be expected to be a potential biomarker of ammonia-lowering therapies in patients with cirrhosis with MHE.
Ahluwalia et al. (124)	*N* = 40 controls *N* = 87 patients with cirrhosis with HE *N* = 67 patients with cirrhosis without HE	Fecal samples	Multitag pyrosequencing	Patients with cirrhosis with HE had a higher relative abundance of *Staphylococcaceae*, *Enterococcaceae*, *Porphyromonadaceae,* and *Lactobacillaceae* compared to controls and patients with cirrhosis without HE.	Specific gut microbial taxa are related to neuronal and astrocytic consequences of cirrhosis-associated brain dysfunction.
Iebba et al. (125)	*N* = 8 patients with cirrhosis with HE *N* = 38 patients with cirrhosis without HE *N* = 14 control	Fecal samples	16S rRNA sequencing	*Bacteroides coprocola* and *Bifidobacterium longum* enhanced the risk of HE, while *Bacteroides faecis* and *Bacteroides coprophilus* lowered the risk of HE.	The feces of patients with liver cirrhosis exhibit functional microecological imbalance, and specific key species are associated with HE.
Sung et al. (74)	*N* = 13 healthy controls *N* = 20 patients with compensated cirrhosis *N* = 15 patients with decompensated cirrhosis *N* = 62 acute HE	Fecal samples	16S rRNA sequencing	*Bacteroidetes* phylum decreased, whereas *Firmicutes*, *Proteobacteria,* and *Actinobacteria* increased in patients with HE compared with those with compensated cirrhosis. Three (*Alistipes*, *Bacteroides*, *Phascolarctobacterium*) and five OTUS (*Clostridium-XI*, *Bacteroides*, *Bacteroides*, *Lactobacillus*, and *Clostridium*-*sedis*) at HE were associated with HE recurrence and overall survival during the subsequent 1-year follow-up.	Gut microbiota may be involved in HE development and able to predict clinical outcomes, providing new strategies for the prevention and treatment of HE recurrence in patients with cirrhosis.
Bajaj et al. (126)	*N* = 29 patients with cirrhosis with MHE *N* = 51 patients with cirrhosis without MHE	Fecal samples	16S rRNA sequencing	There was a lower relative abundance of potentially beneficial taxa (*Lachnospiraceae and Ruminococcaceae*) and higher *Bacteroidaceae* and *Lactobacillaceae* in patients with MHE.	These microbial changes are associated with the diagnosis of MHE.
Bloom et al. (127)	*N* = 33 patients with cirrhosis with OHE *N* = 16 patients with cirrhosis without OHE	Fecal samples	Metagenomic sequencing	*Anaeromassilibacillus species, Anaerostipes caccae, Bacteroides eggerthii, Clostridium species, Faecalicatena contorta, Holdemania filiformis, Neglecta timonensis, and Ruminococcus species* were less abundant in OHE.	Further work is needed to detail this relationship and to develop targeted interventions to treat HE.
Bajaj et al. (128)	*N* = 181 patients with cirrhosis with MHE *N* = 140 patients with cirrhosis without MHE	Fecal samples	16S rRNA sequencing	Patients with MHE had a greater log fold change in *Lactobacillaceae (Pediococcus, Lacticaseibacillus, and Lactobacillus)* and potential pathobionts (*Enterococcus, Klebsiella, Escherichia-Shigella, and Pseudomonas*) compared with those without MHE.	Cognitive impairment in patients with cirrhosis due to HE is related to gut bacterial changes.
Wang et al. (129)	*N* = 30 patients with cirrhosis with OHE *N* = 30 patients with cirrhosis without OHE	Fecal samples	16S rRNA sequencing	Patients with OHE group had higher proportions of *Enterococcus*, *Escherichia-Shigella*, and *Streptococcus* than did patients without OHE.	Reduced microbial species richness and diversity were observed in patients with HE and cirrhosis.
Post-TIPS HE and gut microbiota					
Li et al. (69)	*N* = 28 patients with cirrhosis with MHE	Fecal samples on days 1–3 before surgery and at 1 month after surgery	16S rRNA sequencing	According to the prognosis after TIPS: Non-HE = 8 (*Dialister, Coprococcus, Ruminococcaceae_uncultured, Flavonifractor*, and *Clostridium_sensu_stricto_1* increase). MHE = 12 (*Granulicatella, Enterococcus, Streptococcus*, and *Rothia* decrease and *Veillonella* and *Megasphaera* increase). OHE = 8 (*Veillonella* increase).	The increase in the abundance of native flora may have a certain influence on the remission of patients with MHE.
Li et al. (18)	*N* = 106 patients with cirrhosis	Fecal samples before and after TIPS	16S rRNA sequencing	6 months after TIPS: HE = 33 (autochthonous taxon *Lachnospiraceae* decreased). Non-HE = 73 (autochthonous taxa increased, potential pathogenic taxa decreased). Died = 18 (*Granulicatella, Alistipes,* and lower *Subdoligranulum* before TIPS were the independent risk factors for death).	*Coprococcus, Ruminococcus, Blautia, Ruminococcaceae_uncultured, and Roseburia* were negatively correlated with the severity of HE.
Gitto et al. (71)	*N* = 13 patients with cirrhosis	Fecal samples before and 3 months after TIPS	16S rRNA sequencing	After TIPS, there were increased levels of *Flavonifractor* spp. and decreased levels of *Clostridiaceae*, the latter linked to abdominal infections in cirrhotic patients.	Correction of portal hypertension following TIPS results in modifications of gut microbiota composition could be potentially beneficial and reduce the levels of fecal proinflammatory medium-chain fatty acids.
Zhao et al. (70)	*N* = 30 patients with cirrhosis	Fecal samples before and 1 months after TIPS	16S rRNA sequencing	The abundances of *Haemophilus* and *Eggerthella* increased in patients with HE, whereas those of *Anaerostipes, Dialister, Butyricicoccus,* and *Oscillospira* decreased. The abundances of *Eggerthella*, *Streptococcus*, and *Bilophila* increased in patients without HE, whereas that of *Roseburia* and *Ruminococcus* decreased. Members from the pathogenic genus *Morganella* appeared in patients with but not in patients without HE group.	Intestinal microbiota-related synergism may predict the risk of HE following TIPS in patients with HBV-related portal hypertension. Prophylactic microbiome therapies may be useful for preventing and treating HE after TIPS.

### The inflammatory state caused by dysbiosis of the microbiota is related to post-TIPS HE

Disturbances in the gut microbiota, excessive bacterial growth in the small intestine, and changes in the intestinal barrier can lead to bacterial translocation and further systemic inflammation (75), which is also a major driver of liver cirrhosis-related immune dysfunction (76). Inflammatory reactions play an important role in HE development. Previous studies revealed that the level of endotoxin in the portal vein was the highest in patients with liver disease (77, 78), which indicated that the main source was the intestine and that changes in intestinal bacteria and intestinal permeability can affect the level of endotoxin (79). The reticuloendothelial system (RES) (79, 80) is the main defense system against bacteremia and other infections acquired through blood-borne pathways, with Kupffer cells as the main component. The RES is damaged during liver cirrhosis, and blood bypasses the RES during the portosystemic shunt. Therefore, endotoxemia may worsen after TIPS, which may increase the incidence of HE. One study (81) tested the hypothesis that TIPS exacerbates endotoxemia and reported that 1 h after TIPS, the level of endotoxin in peripheral blood and the brain flux of ammonia increased, but there was no significant change in arterial blood ammonia. Another study (82) also indicated that TIPS can weaken the clearance of endotoxins in the liver after TIPS. However, one study (83) reported that the level of endotoxin in peripheral blood did not increase after TIPS, and it was considered that endotoxemia was due to decreased liver cell function rather than blood shunting. Another study (84) found that patients with alcoholic liver cirrhosis who were on TIPS had similar endotoxins in the portal and hepatic veins during TIPS and decreased portal pressure in 2 weeks after TIPS. No change in endotoxin levels in the portal and hepatic veins indicates that the liver itself may have only cleared a small amount of endotoxin. In addition, one early study reported that (85), after TIPS, the levels of lipopolysaccharide and endothelin in the portal vein are significantly reduced, which can reduce the occurrence of complications, such as endotoxemia caused by intestinal bacterial ectopia.

The differences in the results of the above studies may be due to differences in measurement methods, study time, and patient disease severity, but they also suggests that HE after TIPS may be related not only to portosystemic shunt after stent implantation but also to the intestinal microbiota. Interestingly, hepatic coma and hyperammonemia occur in germ-free animals (86). It was found (87) that hyperammonemia after TIPS may be caused to a large extent by the metabolism of small intestinal cells and has little relationship with intestinal bacteria. Compared with parenteral infusion, enteral infusion had a greater portal ammonia load (29 (21–36) vs. 14 (8–21) mmol/L/240 min) and a higher degree of systemic hyperammonemia (14 (11–17) vs. 9 (6–12) mmol/L/240 min). The small intestinal mucosa extracts glutamine from arterial blood for metabolism by enterocytes and releases considerable quantities of ammonia into the portal vein. Following TIPS, small intestinal ammonia production may lead to a higher degree of systemic hyperammonemia when nutrition is provided via the enteral route rather than the parenteral route. Hence, parenteral nutrition may be superior to enteral nutrition in patients after TIPS because of lower ammonia levels.

Inflammatory changes can not only manifest as endotoxemia but also activate neuroglial cells, induce neuroinflammation, lead to the activation of microglia and a subsequent neuroinflammatory response (88), and cause parenchymal changes in the brain and neurological dysfunction (89). Studies in mice (90) revealed that administration of azoxymethane in mice can reduce the level of IL-6 in plasma and brain tissue, the activation of microglia, and peripheral and cerebral inflammation. Therefore, neuroinflammatory changes may be involved in the occurrence of HE.

**Figure 2:**
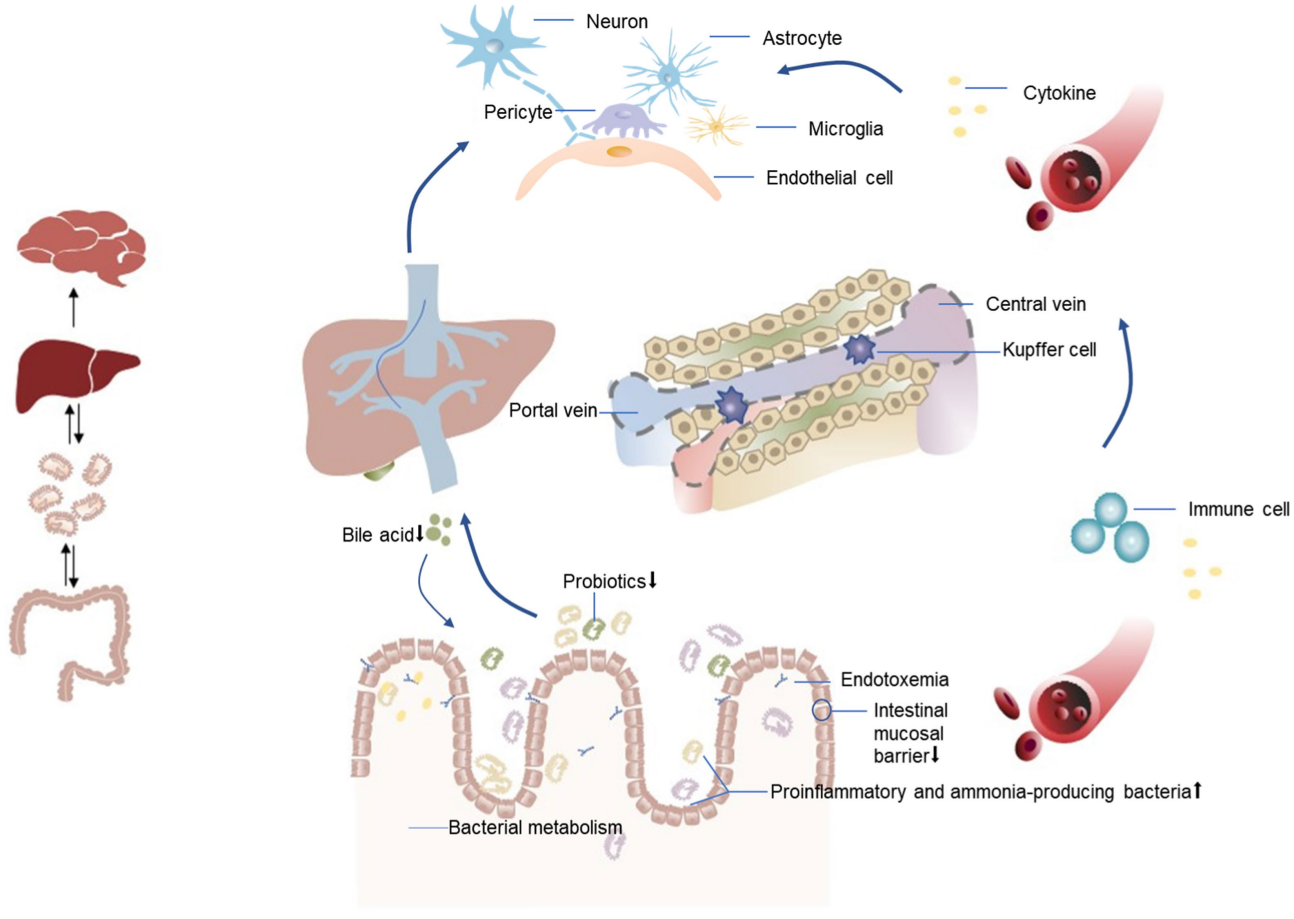
Relationship between the intestinal microbiota and HE after TIPS. An altered gut microbiota in patients with liver disease manifests as a decrease in beneficial bacteria and an increase in harmful bacteria (proinflammatory and ammonia-producing bacteria). This dysbiosis is associated with compromised integrity of the intestinal mucosal barrier, leading to bacterial translocation. Consequently, inflammatory cytokines and toxic substances, including bacterial endotoxins and ammonia, are transported from the intestines to the liver via the portal vein. When liver function is impaired, the ability of Kupffer cells to clear endotoxins is reduced, leading to the entry of endotoxins into the systemic circulation through shunts. Simultaneously, decreased bile acid synthesis promotes bacterial overgrowth, resulting in a systemic inflammatory state. This inflammation may directly trigger neuroinflammation, leading to neurological dysfunction, or facilitate the recruitment of immune cells to brain tissues, ultimately altering brain function.

In addition to the correlations among the intestinal microbiota, inflammation, and HE, metabolites of the intestinal microbiota such as short-chain fatty acids (91) (mainly butyrate, propionate, and acetate), ethanol, bile acids, and choline also affect the metabolism of fat and glucose to varying degrees, affecting the pathophysiology of liver diseases (92) and may also participate in the occurrence of HE (13). For example, bile acids can induce antimicrobial peptides by binding to farnesol X receptors, thereby inhibiting excessive growth of the intestinal microbiota (93, 94). Circulating levels of butyrate were inversely related to portal hypertension, endotoxemia, and systemic inflammation in patients with cirrhosis (95). The relationship between the intestinal microbiota and post-TIPS HE is shown in Figure 2.

## Treatment and prevention strategies for post-TIPS HE

The development of HE after TIPS affected the survival rate in a previous study (32). However, a recent observational study revealed that unlike patients with cirrhosis who did not receive TIPS, episodic OHE after TIPS did not increase the risk of death. However, in patients who underwent TIPS and subsequently died, the incidence of persistent OHE after TIPS was higher (8% vs. 3%) (96). Postoperative HE after TIPS has adverse effects on survival outcomes, and drug strategies for preventing OHE after TIPS remain an unmet need due to the limited evidence of effective preventive treatment.

Current treatment strategies for HE focus primarily on reducing ammonia production and accumulation, inhibiting inflammation, and regulating the intestinal flora (97). The treatment strategy for HE after TIPS is approximately the same as that for common HE (98). MHE and HE Level 1 are generally treated with symptomatic treatment, dietary modification, and medication to prevent further progression of the disease. It is necessary to identify the cause, remove the cause, and strengthen drug treatment for level 2–4 HE. With respect to protein intake (99), in patients with HE Levels 1–2, protein should be limited to 20 g/d in the first few days. With the improvement of symptoms, 10–20 g protein can be added every 2–3 days. Patients with HE Level 3–4 should be prohibited from supplementing protein from the intestine. Plant proteins are superior to animal proteins. Patients who fail to respond to treatment can be treated with stent flow limitation or temporary closure of shunts, artificial extracorporeal liver support can be performed if necessary, and liver transplantation is the ultimate treatment option (99).

However, there is no consensus on whether and which drugs should be used to prevent the occurrence of HE after TIPS. The American and European Clinical Guidelines on HE in Chronic Liver Diseases (100) recommend the use of lactulose to prevent recurrence after the initial onset of HE. The Chinese HE Guidelines (99) mention that lactulose can be used as a preventive drug. According to the European Association for the Study of the Liver (101), lactulose can be used as a secondary preventive measure for HE. Pharmacological prophylaxis is not recommended in North America recommendation for patients who undergo TIPS without a history of HE (102). The BAVENO VII Portal Hypertension Consensus (103) noted that rifaximin can be used for secondary prevention of HE. Currently, there are differences in the effectiveness of drugs in preventing HE after TIPS. In an early study (104) of 75 patients with cirrhosis after TIPS, there was no significant difference in the incidence of HE 1 month after TIPS between the two groups receiving rifaximin or lactulose preventive treatment and without intervention. However, in a recent study (31) involving 197 patients with alcoholic cirrhosis after TIPS, rifaximin prophylaxis reduced the incidence of HE at 6 months after TIPS compared with the non-prevention group (34% vs. 53%; OR 0.48, CI 0.27–0.87). A network meta-analysis (105) aimed to investigate the efficacy of multiple pharmacological regimens for the prevention of post-TIPS HE and revealed that rifaximin alone, lactulose alone, and rifaximin plus lactulose did not significantly reduce the incidence of post-TIPS HE. However, the combination of rifaximin plus lactulose showed the most promising trend toward preventing post-TIPS HE.

Rifaximin is a selective intestinal antibiotic (106). Compared with placebo/no intervention, rifaximin may improve health-related quality of life in patients with MHE. Compared with non-absorbable disaccharides, rifaximin may not have an overall impact on mortality, serious adverse events, health-related quality of life, or HE. However, when used in combination with non-absorbable disaccharides, it may reduce the overall risk of death and the risk of serious adverse events, improve HE, shorten the hospital stay, and prevent the occurrence/recurrence of HE. Therefore, the mechanism by which rifaximin improves cognition may be related to changes in metabolic functions related to the microbiota (107). A study (108) conducted in 20 patients with liver cirrhosis and MHE revealed no significant changes in the fecal microbiota at baseline or 8 weeks after rifaximin 550 mg BID, except for a moderate decrease in *Veillonellaceae* and an increase in *Eubacteraceae*. Another (109) comparison of the feces of patients with liver cirrhosis at baseline and after 4 weeks of rifaximin 400 mg TID also revealed no significant changes in the intestinal composition but a decrease in the relative abundances of the *Veillonella* and *Streptococcus* genera.

Lactulose is a disaccharide composed of galactose and fructose that can be selectively utilized by host microorganisms and is therefore a prebiotic. A study (110) reported that lactulose affects human metabolism and the gut microbiota in a dose-dependent manner. Another study (111) targeting healthy adults revealed that taking 10 g of lactulose daily for 1 month significantly increased the absolute count of the *Bifidobacterium* genus but did not increase the count of other bacteria. After 1 month of treatment with a daily dose of 20 g of lactulose, the counts of cultivable *Bifidobacterium, Lactobacillus,* and *Streptococcus* increased, whereas the counts of *Bacteroides, Clostridium, Escherichia coli*, and *Eubacterium* decreased (112). Interestingly, unlike healthy individuals, after intervention with lactulose, the gut microbiota of patients with liver cirrhosis did not change, indicating that the impact of lactulose on HE may not be related to changes in the gut microbiota (113). However, there is still a lack of research regarding the efficacy of intestinal microecological agents in the prevention or treatment of HE following TIPS. A summary of the literature on drug prevention of post-TIPS HE is provided in Table 2.

**Table 2:** Study of drug prevention of post-TIPS HE.

References	Subgroup	Research objective	Research results
Riggio et al. (104)	*N* = 25 (Lactulose 60 mg QD) *N* = 25 (Rifaximin1200 mg QD) *N* = 25 (No treatment)	The incidence of OHE at 1 month after TIPS.	The incidence of HE in the lactulose group, rifaximin group, and control group was 36, 32, and 32%, respectively (*p* = 0.97).
Bai et al. (130)	*N* = 21 (L-ornithine-L-aspartate, LOLA) *N* = 19 (Control)	To evaluate the effects of LOLA on venous ammonia after TIPS.	The use of LOLA after TIPSS can significantly reduce the increase of venous ammonia and benefit the patient’s mental status.
Riggio et al. (131)	*N* = 23 (Albumin) *N* = 45 (Control)	To study whether albumin infusion can influence the HE occurrence and the blood ammonia level at 1 month after TIPS.	No differences in the incidence of OHE, venous blood ammonia levels, and psychometric tests between groups.
Subramanian et al. (132)	*N* = 27 (No prophylaxis) *N* = 38 (Lactulose) *N* = 6 (Rifaximin) *N* = 73 (Lactulose and Rifaximin)	To assess the survival benefit of HE prophylaxis.	The use of lactulose and/or rifaximin for treating patients with HE prophylaxis increases survival 12 months after TIPS. The survival effect is more dramatic among patients without a prior diagnosis of HE or who received previous treatment for HE.
Seifert et al. (118)	*N* = 83 (Untreated) *N* = 85 (Lactulose, dose varies from person to person, defecation BID/TID) *N* = 6 (Rifaximin, 550 mg BID) *N* = 59 (Lactulose + Rifaximin)	Analyze the risk factors for HE after TIPS. Assess the effectiveness of preventive drug treatment.	Age and pre-TIPS HE history are risk factors for post-TIPS HE. Lactulose has no preventive effect. Compared with lactulose and no treatment, rifaximin combined with lactulose can prevent the recurrence of HE at 12 months after TIPS in patients with a previous history of HE (25.0% vs. 64.7%, *p* = 0.007), but it cannot prevent the occurrence of postoperative HE in patients without a history of HE.
Bureau et al. (31)	*N* = 97 (Rifaximin, 600 mg BID) *N* = 100 (placebo)	Occurrence of HE within 168 days after TIPS.	Rifaximin can effectively prevent the occurrence of HE 168 days after TIPS (35.3% vs. 55.5% *p* = 0.008).

QD, one time per day; BID, two times per day; TID, three times per day.

## The significance of intestinal microbial agents in preventing post-TIPS HE

Intestinal microecological therapy is a method of treating diseases by restoring normal flora, inhibiting pathogenic bacteria, and promoting microecological balance and includes mainly microecological agents (probiotics, etc.) and fecal flora transplantation therapy (114). A meta-analysis based on multiple randomized controlled trials (115, 116) revealed that the use of probiotics in patients with HE could reduce hospitalization rates, improve CHE, and prevent progression to OHE. A randomized trial conducted in patients with recurrent HE (117) revealed that compared with standard treatment, fecal flora transplantation could reduce readmission rates, improve patient cognition, and increase flora diversity. Research on the prevention or treatment of HE after TIPS is limited, and interventions are mostly lactulose, rifaximin, or ornithine aspartate (118, 119). There are limited studies and inconsistencies in the findings. It is unclear whether intestinal microecological intervention after TIPS affects changes in the intestinal flora and the relationship between such changes and postoperative HE. In a case report on the application of fecal microbial transplantation in patients with HE after TIPS (120), beneficial bacteria such as *Ruminococcus* decreased in patients with HE after TIPS, whereas harmful bacteria and opportunistic pathogenic bacteria such as Veillonella increased. After fecal microbial transplantation, changes in the intestinal flora of patients were significant. There were no further hospital admissions for HE during the 1-year follow-up period. Therefore, intestinal microecological therapy may be a new treatment for preventing HE after TIPS.

*Clostridium butyricum* (121) is an inherently beneficial bacterium that has been proven to have potential protective or beneficial effects on the body in patients with inflammatory bowel diseases, neurodegenerative diseases, metabolic diseases, and colorectal cancer. Previous studies have shown that beneficial bacteria such as *Clostridium butyricum* are significantly reduced in patients with liver cirrhosis (122). *Clostridium butyricum*, also known as *Clostridium butyricum*, can inhibit pathogenic bacteria and promote the growth of beneficial bacteria in the intestine. It can convert lactic acid to butyric acid, accelerate mucin production, reduce propionic acid and acetic acid levels, and prevent the destruction of epithelial mucin, thereby maintaining a healthy intestinal tract and enhancing its barrier effect. It has been found that (123) *Clostridium butyricum* can reduce the expression of Toll-like receptor 4 in colon epithelial cells, and inhibiting the activation of NF-κB signaling pathway can reduce the expression of lipopolysaccharide-induced proinflammatory cytokines, alleviate intestinal mucosal damage, and thereby reduce the level of endotoxin in the body, which can delay the progression of liver fibrosis. There is still a lack of literature on whether it can reduce the incidence of HE after TIPS. Research on the prevention or treatment of HE with probiotics is shown in Table 3. In recent years, studies have also begun to explore the efficacy of fecal microbiota transplantation in the treatment of recurrent HE, but this research is still in clinical trials. Research has shown that FMT has good safety and tolerability, but the efficacy of treating HE varies from person to person. We have also added this information to the literature after (Table 3).

**Table 3:** Probiotics used for the treatment or prevention of HE.

References	Subgroup	Probiotics	Research objective	Research results
Sharma et al. (133)	*N* = 190 cirrhosis without OHE (*N* = 35 Lactulose; *N* = 35 Probiotics; *N* = 35 Lactulose and Probiotics)	Dose 1 capsule three times/day for 1 month (each capsule contained *Streptococcus faecalis* 60 million, *Clostridium butyricum* 4 million, Bacillus mesentericus 2 million, and lactic acid bacillus 100 million)	Compared lactulose with probiotics and a combination of lactulose plus probiotics in the treatment of MHE.	Lactulose and probiotics separately or in combinations are equally effective in the treatment of MHE.
Mittal et al. (134)	*N* = 160 cirrhosis with MHE (*N* = 40 Lactulose; *N* = 40 Probiotics; *N* = 40 L-ornithine-L-aspartate; *N* = 40 Control)	Probiotics (110 billion colony-forming units twice a day) for 3 months	To compare lactulose, probiotics, and L-ornithine L-aspartate for the treatment of MHE and their effect on daily functioning and health-related quality of life.	14 patients (35%) with MHE recovered in the probiotic group. 2 patients (5%) developed OHE. No significant improvement in quality of life compared to the control group.
Saji et al. (135)	*N* = 43 cirrhosis (*N* = 21 Probiotics; *N* = 22 Control)	Probiotic preparation in a one gram sachet containing not less than 1.25 billion spores of *Lactobacillus acidophilus, Lactobacillus rhamnosus*, *Bifidobacterium longum* and *Saccharomyces boulardii*, three times daily after meals	To assess the efficacy of probiotics for treating MHE in patients with cirrhosis.	There was no statistically significant change in parameters such as arterial ammonia, evoked responses, and number of connection tests before and after treatment with probiotics compared to placebo.
Lunia et al. (136)	*N* = 160 cirrhosis without OHE (*N* = 84 Probiotics group; *N* = 74 Control)	Probiotics (1 × 10^8^ colony-forming units, 3 times daily) for 3 months	Study the efficacy of probiotics in the primary prevention of HE. The main endpoint is the development of explicit HE.	Compared with baseline, probiotic significantly reduced levels of arterial ammonia, small intestinal bacterial overgrowth, and orocecal transit time; increased psychometric HE scores; and increased critical flicker fusion thresholds. Seven patients in the probiotic group and 14 controls patients developed OHE (*p* < 0.05; HR = 2.1; CI 1.31–6.53).
Ziada et al. (137)	*N* = 90 patients with MHE (*N* = 30 Lactulose; *N* = 30 probiotic; *N* = 30 Control)	Probiotic (one capsule containing 106 *L. acidophilus* three times/day)	To evaluate probiotics as alternative therapy for treating MHE.	The relative risk reduction of developing OHE was 60% in the patients receiving lactulose and 80% in patients receiving probiotic.
Sharma et al. (138)	*N* = 124 cirrhosis with MHE (*N* = 31 L-ornithine-L-aspartate; *N* = 31 Rifaximin; *N* = 32 Probiotic; *N* = 30 Control)	400 mg thrice a day for 2 months (*Lactobacillus acidophilus* 0.7 billion, *Lactobacillus rhamnosus* 0.6 billion, *Lactobacillus plantarum* 0.6 billion, *Lactobacillus casei* 0.6 billion, *Bifidobacterium longum* 0.6 billion, *Bifidobacterium infantis* 0.6 billion, *Bifidobacterium breve* 0.6 billion, *Saccharomyces boulardii* 0.1 billion, and *Streptococcus thermophilus* 0.6 billion)	To determine the effect of rifaximin, probiotics, and l-ornithine l-aspartate (LOLA) individually in reversal of MHE by comparing it with placebo group.	The number of patients who improved after treatment was 67.7% (21/31), 70.9% (22/31), 50% (16/32), and 30% (9/30) in the groups receiving LOLA, rifaximin, probiotics, and placebo, respectively.
Mouli et al. (139)	*N* = 77 chronic liver disease with MHE (*N* = 40 Lactulose; *N* = 33 Probiotic)	Probiotic (four capsules of VSL#3; total of 450 billion CFU/day) for 2 months	To test whether probiotics are non-inferior to lactulose in improving MHE.	MHE improved in 25 (62.5%) patients taking lactulose and 23 (69.7%) taking probiotics.
Fecal microbiota transplant (FMT) and HE				
Bajaj et al. (117)	*N* = 10 FMT *N* = 10 Control	Three frozen-then-thawed FMT units (90 mL total) were administered by enema and retained for 30 min	Aimed to define whether FMT using a rationally derived stool donor is safe for treating recurrent HE compared to standard of care (SOC).	Eight patients (80%) receiving SOC participants had a total of 11 SAEs, while those receiving FMT had two (20%) participants with SAEs (*p* = 0.02). Cognition improved in patients receiving FMT but not in those receiving SOC. FMT increased in relative abundance of *Lachnospiraceae* and *Ruminococcaceae*.
Bajaj et al. (140)	*N* = 10 FMT (1 died) *N* = 10 Control (1 died, 1 underwent liver transplantation)	Three frozen-then-thawed FMT units (90 mL total) were instilled by enema and retained for 30 min	Aimed to determine the long-term impact of FMT on cognition, hospitalizations, and HE episodes by extending the results of this trial.	There were a significantly higher number of hospitalizations and HE episodes in patients receiving SOC arm compared to patients receiving FMT during the long-term follow-up.
Bajaj et al. (141)	*N* = 15 FMT capsules *N* = 15 Control	Patients were randomly administered 15 capsules of placebo or FMT	Aimed to determine the safety, tolerability, and impact on mucosal/stool microbiota and brain function in patients with HE after capsular FMT.	Fecal capsules are safe and well tolerated. Capsule fecal microbiota transplantation reduced readmission rate but did not decrease HE episodes. After FMT, the diversity of duodenal mucosa increased, cytokines and barrier proteins changed, and LBP levels decreased.
Bloom et al. (142)	*N* = 10 FMT capsules	On average, 15 capsules contained 24 g of fecal matter. Patients were administered 15 oral FMT capsules on days 1, 2, 7, 14, and 21	Primary outcomes were change in psychometric HE score (PHES) and serious adverse events (SAEs).	FMT capsules improved cognition in patients with HE, with an effect varying by donor and recipient factors. PHES improved 4 weeks after the fifth dose of FMT.
Bajaj et al. (143)	Group 1 = 15 (3 active and 0-placebo) Group 2 = 15 (2 active and 1-placebo) Group 3 = 15 (1 active and 2-placebo) Group 4 = 15 (3-placebo)	Subjects received three administrations (60 mL enema and five capsules at baseline and five more capsules at day 30)	FMT-related serious adverse events. Secondary outcomes were HE recurrence, all-cause hospitalizations, death, donor engraftment, and quality of life.	FMT-related SAEs were not observed in any of patients receiving FMT groups. HE recurrence was highest in the patients in the group all receiving placebo vs. in patients in the group all receiving FMT (40% vs. 9%) (associated with lower baseline *Lachnospiraceae* and reduced donor engraftment.). Within patients receiving FMT, HE-recurrence rates were similar.

Group 1 (receiving 3 active capsules and 0-placebo): active capsule and enema FMT at baseline and active capsules at day 30; Group 2 (receiving 2 active capsules and 1-placebo): active capsule at baseline and day 30 and placebo enema at baseline; Group 3 (receiving 1 active capsules and 2-placebos): active enema at baseline and placebo capsules at baseline and day 30; Group 4 (receiving 3-placebos): placebo capsules and enema at baseline and placebo capsules at day 30.

## Conclusion

HE after TIPS affects the quality of life of patients and their families, and there are currently no effective preventive measures. The intestinal microbiota has been shown to be associated with the occurrence and prognosis of various diseases. The intestinal microbiota has become increasingly widely used in clinical practice, and restoring intestinal homeostasis is one of the goals of liver disease treatment. The predictive value of the intestinal microbiota in post-TIPS HE and the significance of intestinal microecological agents in the prevention and treatment of post-TIPS HE require further study. In summary, the intestinal flora has a promising future in the occurrence and treatment of HE after TIPS.
